# The Effect of Food Polymers (Pectin, Alginate, and Gum Arabic) on Carbonated Drink-Induced Enamel Demineralization: An In Vitro Study

**DOI:** 10.7759/cureus.56385

**Published:** 2024-03-18

**Authors:** Jinu George, Jolly Mary Varughese, Subija Kunnumpurath Narayanankutty, Divia Attuvalappil Rajan, Dhanya Shaji, Christalin Ramakrishnan

**Affiliations:** 1 Department of Conservative Dentistry and Endodontics, Government Dental College, Thrissur, IND; 2 Department of Conservative Dentistry and Endodontics, Government Dental College, Trivandrum, IND

**Keywords:** laser-induced fluorescence, carbonated drinks, food-approved polymers, scanning electron microscopy, enamel demineralization

## Abstract

Introduction: The increased use of soft drinks leads to a high prevalence of dental erosion (DE), and the use of polymers can decrease tooth demineralization by a carbonated drink. Assessment of the effect of food-approved polymers such as highly esterified pectin (HP), propylene glycol alginate (PGA), and gum arabic (GA) on their efficiency to reduce enamel demineralization on addition with a commercially available carbonated drink was the main objective of this study.

Materials and methods: For this study, 300 premolar teeth were studied for enamel erosion and were divided into five groups consisting of 60 samples in each group. The teeth treated with distilled water had negative control, a commercially available carbonated drink with pH 2.7 had positive control, and food polymers were added individually to the carbonated drink in a specified quantity with minimal pH change and were taken as groups A, B, and C, respectively. The enamel erosion that occurred in study groups was measured using a laser fluorescence spectroscopic system with laser excitation at 404 nm at different treatment times (30, 60, and 120 seconds).

Results: Demineralization was less in samples treated with polymer added to carbonated drink solutions compared to samples exposed to plain carbonated drink. As the time of exposure increased up to 120 seconds, a significant decrease in demineralization occurred in polymer-treated groups of samples as against plain carbonated drink with HP showing more decreased demineralization with extended exposure periods compared to other polymers. The surface morphology of tooth samples exhibited the anti-erosive effect of polymers, and the scanning electron microscopic pictures revealed a smoother surface for the polymer-added group.

Conclusion: This study shows the efficacy of HP, PGA, and GA on reducing the effect of carbonated drink-induced enamel demineralization, and these polymers' addition to drinks can be an innovative way to reduce the demineralization potential of carbonated acidic drinks.

## Introduction

Dental demineralization is caused by the close interaction of an acid with the tooth surface, and the main etiologic factors can be chemical, environmental, or behavioral. We have been observing that the frequency of dental caries has been decreasing over time. However, there has been a significant increase in the incidence of noncarious lesions globally. This changing trend can be attributed to the increased use of soft drinks and confectionery, resulting in demineralization of enamel or dental erosion (DE) [1]. DE is a surface-softening lesion that is susceptible to tooth wear. These lesions are often shown to affect the entire dentition. New diet regimens and lifestyle changes have been shown to contribute highly to the occurrence of erosive tooth lesions. If these lesions are not managed timely and effectively, they can cause considerable degradation of the enamel and eventually lead to exposure of the underlying dentin, which leads to dentinal sensitivity, reduction of vertical height, and esthetic concerns [2].

Soft drinks and fruit juices often have a pH of less than 4.5 and, when in proximity to the tooth, tend to decrease the pH of the tooth surface below the critical pH of 5.5, resulting in its demineralization [3]. Even acidic center-filled chewing gums and vitamin C tablets have been shown to have an erosive effect on the tooth surface [4,5]. Though erosive changes can be managed restoratively, further exposure to erosive challenges may cause the failure of these restorations due to marginal deterioration and continued loss of surrounding dental hard tissue. Thus, an individual’s dietary habits form an important factor in determining one’s susceptibility to DE.

DE is a pathological condition characterized by the persistent and localized loss of hard dental tissues from the tooth surface. This loss occurs through a chemical process, without the active involvement of microbes [6]. There is a variance in the prevalence of DE in children and adolescents around the world, with rates varying from 7.2% [7] to 95%. [6] It has shown a clear increase with age and is emerging to be a common condition limited to developed countries [8]. Preventive measures should hence be adopted not only for early intervention and primary prevention but also for secondary prevention and preservation of restorations. Diet modifications and altered compositions for soft drinks are areas that need to be emphasized to change the scenario, and hence, food-approved polymers are being investigated for their potential to reduce the demineralization of acidic drinks [9]. New formulations for "tooth-friendly soft drinks," exhibiting a considerably reduced erosive propensity than traditional soft drinks could be introduced into the market following proper research.

Visual and tactile examinations are used conventionally to identify demineralization, but they are unable to diagnose very early/incipient lesions. The other reliable methods are electronic monitoring (ECM), fiber-optic transillumination (FOTI), and DIAGNOdent [10]. The conventional diagnostic techniques can be used in cavitated lesions but are not enough for the detection of the noncavitated ones. These techniques have drawbacks and lack feasibility in a clinical setting. Laser-induced fluorescence spectroscopy (LIFS) is a highly effective method for detecting and measuring demineralization, allowing for the identification of even the earliest stages of caries and demineralization. The demineralization process leads to a reduction in the intensity of the fluorescence signal. This is caused by the degradation of the enamel layer's prism configuration and the loss of its waveguide capabilities. The LIFS technique can be employed to identify these changes [11].

As the consumption of carbonated drinks is increasing in day-to-day life, reduction of their erosive potential is a practical and responsible way to minimize the risk. The decrease in erosion by the process of proteins found in milk adhering to tooth enamel has been documented [12]. Several food-grade polymers have been studied to determine their ability to decrease the breakdown of calcium and phosphate in enamel. Polymers such as highly esterified pectin (HP), propylene glycol alginate (PGA), and gum arabic (GA) are commonly employed in confectionery, jams, bakery goods, and similar items as preservatives, foam stabilizers, and thickening agents. When included in carbonated beverages, these polymers combine with hydroxyapatite (HA) and form a protective coating on the surface of the enamel [13]. Thus, the addition of these polymers to drinks can be an easy way to reduce the demineralization potential of carbonated drinks. This in vitro study was conducted on extracted premolars to study the effect of intentionally added polymers such as HP, PGA, and GA to the extent of enamel demineralization caused by a commercially available carbonated drink using a non-destructive LIFS and scanning electron microscopy (SEM).

## Materials and methods

Three hundred sound and vital premolars extracted for orthodontic purposes were collected. The study was conducted at the Government Dental College, Trivandrum, after getting ethical approval (IEC/IRB No. IEC/C/RP-31/2012/DCT/ dated 11-12-2012). Teeth extracted from patients with systemic/genetic/developmental conditions affecting mineralization of dentition and teeth with surface defects like caries, hypoplasia, erosion, attrition, etc., were excluded from the study. Universal guidelines for handling extracted teeth according to the Centers for Disease Control and Prevention (CDC) were undertaken. Roots were resected using a diamond disc (P-129 Diamond cutting disc, 18 mm diameter) in a straight handpiece. The crown was sectioned longitudinally in a buccolingual direction to obtain two halves, and the cut surface consisted of an inner layer of dentin and an outer layer of enamel. The laser probe was precisely placed on this outer layer of enamel to record the typical enamel fluorescence without the interference of dentin. The specimens were stored in deionized water for further use.

A commercially available carbonated drink was taken as a positive control. The pH of the drink was determined using a pH meter and was 2.7. Distilled water was taken as a negative control. Three test solutions were prepared by dissolving 1 wt% of polymers: HP, PGA, and GA (separately in deionized water). Polymer powders were weighed in an electronic weighing machine (AUX 220 Analytical Balance, Shimadzu Corporation, Kyoto, Japan). Ultrasonic desiccators were used for 15 minutes to aid in the dissolution of polymers in deionized water. To obtain polymer-modified carbonated drink, each of the polymer solutions was added to the carbonated drink so that the final solution would have 1 wt% polymer, without significant change in pH. About 30 mL of each solution was used for each sample. The teeth specimens were divided into five groups with each group of 60 samples (Table 1).

**Table 1:** Study groups based on the treatment given

Groups	Number of teeth	To be immersed in 30, 60, and 120 seconds
Group A	60	Carbonated drink + highly esterified pectin (HP)
Group B	60	Carbonated drink + propylene glycol alginate (PGA)
Group C	60	Carbonated drink + gum arabic (GA)
Group D (positive control group)	60	Carbonated drink alone
Group E (negative control group)	60	Distilled water alone

Samples in each group were treated with their respective test solutions for 30, 60, and 120 seconds, respectively, and were constantly agitated during treatment. The study groups are divided as follows: Group A, carbonated drink with HP; Group B, carbonated drink with PGA; Group C, carbonated drink with GA; Group D, carbonated drink alone; and Group E, distilled water alone. The samples were then rinsed in deionized water for 30 seconds and dried using blotting paper. The stimulation beam for laser-induced fluorescence experiments is generated by a diode laser (Stocker Yale Inc., Canada; Model: TEC-XXX-404S-50-SF) that emits light at a wavelength of 404 nm. The diode laser was linked to an optical cable (Ocean Optics Inc., USA; BIF 200 UV VIS) that directs the radiation to a 3-meter-long reflection probe (Ocean Optics Inc., USA; ZR400-5 VIS-NIR). The probe is capped with a stainless-steel ferrule measuring 15 cm long and 6 mm in width.

This reflection probe has a central illumination and six pick-up fibers, all with an average diameter of 400 μm. These fibers are used for collecting and transferring fluorescence from the dental enamel to a small fiber-optic spectrometer (Ocean Optics Inc., USA; Model: USB2000-FL). During fluorescence experiments, the light that emanates from the tissue is filtered using a long-wavelength pass filter (Schott GG420) that is placed in the inline filter holders (Ocean Optics Inc., USA; FHS-UV). This filter prevents the stimulated laser beam that is scattered backward from accessing the spectrometer. The spectrometer is linked to the USB connection of a personal computer to manage the process of acquiring spectral information and storing it. To prevent the entry of natural light into the detecting system, a pliable, elongated black PVC cover was positioned at the tip of the probe.

The specimens from every group were affixed to wax molds for convenient manipulation, with the sliced side facing upward. The samples were stimulated using laser energy by positioning a fiber-optic light probe on the exterior enamel surface of the specimens, specifically on the buccal side. The alignment of the optical fiber light couplings was adjusted to deliver a Gaussian beam pattern at the leading edge of the fiber, and the power output was then evaluated. The tip of the probe was gently pressed to prevent any displacement of the object from being detected and to prevent any interference from ambient light from reaching the detecting system. The spectrum of the emitted light was captured using a spectrometer equipped with a 2048-element linear silicon charge-coupled device (CCD) array of data. The range of wavelengths covered was from 400 to 750 nm. The data was captured using the Ocean Optics Base32 software, Version 2.0.0.3 (Ocean Optics, Inc., USA). The software was set up to calculate an average of 40 scans, with a boxcar width of 10 nm and an integration duration of 100 ms. Each specimen was probed to get a minimum of 15 readings. The spectrum of fluorescent substances was taken from 30 specimens in every group using the same method. The fluorescence intensity is believed to have an inverse correlation with demineralization. Demineralization increases as fluorescence intensity decreases and vice versa.

A total of five premolars were retrieved. When treating extracted teeth, universal safety precautions were followed, as advised by the CDC. The roots were excised using a P-129 Diamond cutting disc with a diameter of 18 mm attached to a straight handpiece. The crown was divided into two halves along its buccolingual axis, and one-half of each tooth was chosen for the study. Depending on the research groups, every tooth specimen was submerged and shaken for 120 seconds. The samples were subsequently dried using ethanol concentrations that increased over time, such as 50%, 70%, and 90% for 20 minutes each, and 100% for 60 minutes. The specimens were placed on absorbing paper and left to desiccate in a sealed container overnight. After undergoing critical point drying, the samples were affixed to a specimen holder using double-faced stickers and covered with gold using a process called sputter coating in a vacuum. They were then studied utilizing a JEOL Model JSM-6390LV SEM.

The gathered raw data was summarized using descriptive statistics, including the arithmetic mean and standard error (SE). The data was compiled using MS Excel (Microsoft Corporation, Redmond, Washington, United States). The data was analyzed using the IBM SPSS Statistics for Windows, Version 26.0 (Released 2019; IBM Corp., Armonk, New York, United States) to compare the study groups in terms of fluorescence intensity. The collected data on fluorescence intensities of tooth samples during 30, 60, and 120 seconds treatment among groups A, B, C, D, and E were subjected to statistical analysis using one-way analysis of variance (ANOVA). If ANOVA shows significance, pair-wise comparisons between groups are carried out by using a posthoc least significant difference (LSD) test. A p-value that is less than 0.05 is deemed statistically significant at a 5% significance level.

## Results

The fluorescence intensity was inversely related to demineralization. Demineralization increases over time, and the fluorescence intensity was observed as reduced. The spectral features show a decrease in fluorescence intensity indicating an increase in demineralization and vice versa. With the increased treatment time, the fluorescence intensity was observed to be increased, which means the demineralization decreased.

**Figure 1:**
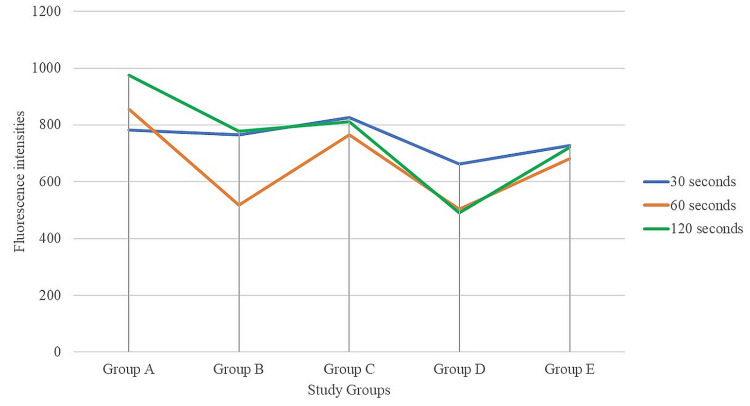
The groupwise comparison of the demineralization among the study groups at 30, 60, and 120 seconds

The recorded spectra were normalized to maximum fluorescence emission intensity to compare between groups and whether there is any difference due to demineralization in the spectral profile. A major difference is noticed in the spectral profile when the tooth surface is demineralized for 120 seconds. The spectra at 120 seconds showed a definite redshift (a shift to the right) in the emission profile. Therefore, the intergroup comparison graph is shown at 120 seconds in Figure 1.

There was a shift to the right for carbonated drinks. This indicates that greater demineralization occurred when samples were treated with carbonated drinks for 120 seconds compared to other groups.

The statistical treatment of the collected data on fluorescence intensity among different study groups at different time intervals has been presented in Table 2.

**Table 2:** Intergroup comparison concerning fluorescence intensity at different time intervals (30, 60, and 120 seconds) SE: standard error Intergroup comparison concerning fluorescence intensity was done using a one-way analysis of variance (ANOVA). Unit of fluorescence intensity is expressed in arbitrary units (AU).

Groups	30 seconds		60 seconds		120 seconds	
	Mean	SE	Mean	SE	Mean	SE
A	782.47	42.70	854.62	27.54	974.50	40.18
B	765.22	40.78	517.63	46.76	777.83	52.40
C	825.28	45.43	766.13	41.06	810.46	44.27
D	663.12	25.32	503.97	26.74	490.43	36.81
E	728.08	35.02	682.07	34.02	722.21	36.12
p-value	0.040		<0.01		<0.01	

The collected data on fluorescence intensities of tooth samples during 30, 60, and 120 seconds treatment among groups A, B, C, D, and E were subjected to statistical analysis using ANOVA. Descriptive statistics such as arithmetic mean, SE, and p-value were calculated to summarize the raw data. If ANOVA shows significance, pair-wise comparisons between groups are carried out by using a posthoc LSD test.

At 30 seconds, the statistically significant mean difference exists among groups for fluorescence intensity (p < 0.05). The polymer-modified carbonated drink solution groups (A, B, C) showed a significant increase in intensity from groups D and E. The intensity in Group E was significantly lower than that in Groups A, B, and C and significantly higher than that in Group D. Group D showed significantly lower intensity compared to other groups. Groups A, B, and C showed no statistically significant difference among the groups for fluorescence intensity.

At 60 seconds, statistically significant mean difference among groups concerning fluorescence intensity (p < 0.01). The polymer-modified carbonated drink solution groups (A, C) showed significantly higher fluorescence intensity than groups D and E at 60 seconds. Group D showed significantly lower fluorescence intensity compared to the other groups. Groups A and C showed statistically significant differences with Group B for fluorescence intensity. Group B was not statistically significant from Group D.

At 120 seconds, statistically significant mean difference exists among groups for fluorescence intensity (p < 0.01). The polymer-modified carbonated drink solution groups (A, B, C) showed a significant difference in fluorescence intensity compared to Groups D and E at 120 seconds. Groups A, B, and C showed increased fluorescence intensity compared to groups D and E. Group D showed significantly lower fluorescence intensity than the other groups. The intensity of Group E was significantly lower than that in Groups A, B, and C and significantly higher than that in Group D. Groups A and C showed no statistically significant difference between the groups for fluorescence intensity. Group B showed a significant decrease in intensity compared to groups A and C.

Comparing the fluorescence intensities of the five groups at three different treatment periods, the polymer-modified carbonated drink solutions showed greater fluorescence intensity indicating lesser demineralization compared to carbonated drink. Demineralization by carbonated drinks increased with the exposure period. The demineralization-reducing effect of polymer-modified solutions showed an increase with time of exposure. At 120 seconds, polymer-added solutions showed increased intensities indicating lessened demineralization.

ANOVA was again used to compare the mean differences in fluorescence intensity at 30, 60, and 120 seconds within each group (Table 3).

**Table 3:** Intragroup comparison of fluorescence intensities at different time intervals Intragroup comparison of fluorescence intensities was done using a one-way analysis of variance (ANOVA) test. Unit of fluorescence intensity is expressed in arbitrary units (AU).

Groups	A		B		C		D		E	
Time (in seconds)	Mean	SE	Mean	SE	Mean	SE	Mean	SE	Mean	SE
30	782.5	40.7	765.2	42.7	825.3	45.4	663.1	25.3	728.1	35.0
60	854.6	46.76	517.6	27.54	766.1	41.06	504.0	26.74	682.1	34.02
120	974.5	52.40	777.8	40.1	810.5	44.27	490.4	36.81	722.2	36.12
p-value	<0.01		<0.01		>0.05		<0.01		>0.05	

ANOVA showed a statistically significant mean difference exists among Group A for fluorescence intensity (p < 0.01) at 30, 60, and 120 seconds. The polymer (HP)-modified carbonated drink solution Group A showed a statistically significant difference in fluorescence intensity at 30, 60, and 120 seconds. The fluorescence intensity increases as time increases indicating that the rate of demineralization decreases as time of exposure increases.

ANOVA showed that a statistically significant mean difference exists among Group B for fluorescence intensity (p < 0.01) at 30, 60, and 120 seconds. The polymer (propylene glycol alginate)-modified carbonated drink solution Group B showed a statistically significant difference in fluorescence intensity at different time exposures. There was no statistically significant difference at 30 and 120 seconds. The fluorescence intensity decreases initially as time increases to 60 seconds but later increases at 120 seconds. There was no statistically significant mean difference among Group C for fluorescence intensity (p > 0.05). The polymer (GA)-modified carbonated drink solution Group C showed no statistically significant difference in fluorescence intensity at 30, 60, and 120 seconds.

The statistically significant mean difference exists among Group D concerning demineralization (p < 0.01). The plain carbonated drink solution Group D showed a statistically significant difference in demineralization at 30, 60, and 120 seconds. The fluorescence intensity decreased as time increased indicating increased demineralization. ANOVA showed that no statistically significant mean difference exists among Group E for fluorescence intensity (p > 0.05). Distilled water Group E showed no statistically significant difference in intensity at 30, 60, and 120 seconds.

Pectin-added solution showed decreased demineralization of enamel samples in 30, 60, and 120 seconds, and this demineralization-reducing effect increased with time. PGA-modified drink decreased demineralization in 30, 60, and 120 seconds. GA showed decreased demineralization at all-time exposures. This shows that these polymers are effective in reducing the induced demineralization. The carbonated drink group showed decreasing fluorescence intensities, indicating increasing demineralization as time progressed.

**Figure 2:**
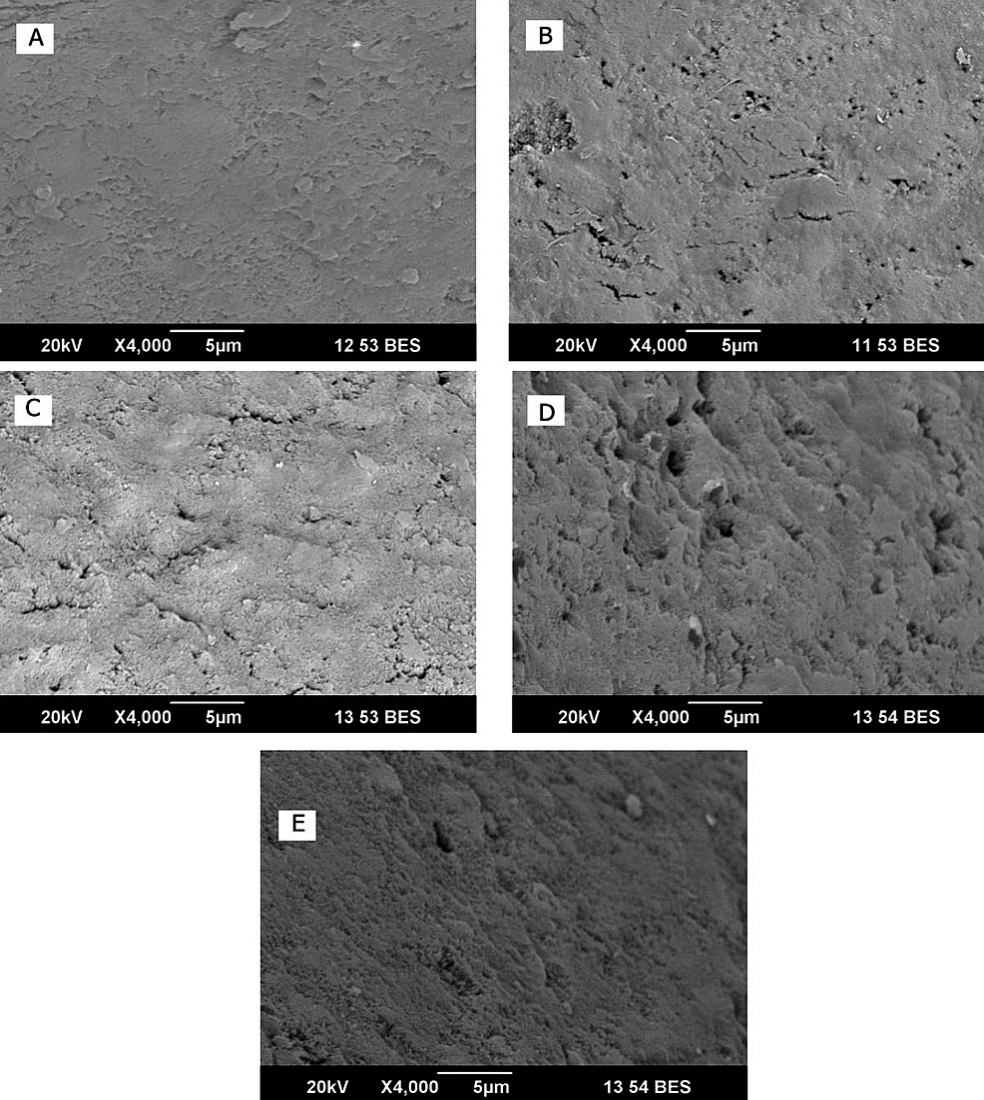
Scanning electron microscopy (SEM) images of the groups A) Group A showed surface features of the enamel exposed to highly esterified pectin-added carbonated drink, with a smooth uniform texture indicating a layer of polymer on the enamel surface. B) In Group B, where propylene glycol alginate (PGA) was used, a moderate number of dentinal tubules were visible. The layer of polymer coating was not uniform, with uneven distribution. C) Group C showed a small number of dentinal tubules, and an irregular polymer surface layer was observed when exposed to gum arabic. D) Group D showed surface morphology of the enamel sample after exposure to carbonated drink alone. There was an increase in surface porosities, scalloping of surface, and opening of dentinal tubules. E) In Group E, where the enamel surface was treated with distilled water, the surface was not completely smooth. A uniform aprismatic surface layer can be observed.

One sample from each group after being treated with the solutions for 120 seconds was examined under SEM. Scanning photomicrographs were obtained at 4000× magnification at 20 kV with a scanning electron microscope on qualitative interpretation of micrograph with a depth of focus of 5 μm. The enamel characteristics were also similar at 2 μm depth of focus (Figure 2).

On polymer-modified carbonated drink exposure, the enamel surface appeared to be smooth, which could be due to the formation of a layer of polymer on the enamel surface. Carbonated drink-exposed enamel appeared to be irregular with the opening of dentinal tubules due to enamel erosion.

## Discussion

Studies based on the use of food-approved polymers in carbonated drinks for erosion reduction are limited. In previous studies, the effect of these polymers on erosion was investigated in a citric acid solution [14]. Previous studies have employed profilometry for quantification of the effect of different polymers on the erosive potential of citric acid solutions [15]. An experimental acidic soft drink with polyphosphate and gum was found to be less erosive than with polyphosphate alone [16]. To the best of our knowledge, the effect of the addition of food polymers directly to a commercially available carbonated drink has not yet been investigated. In the present study, the pH of the drink was only minimally changed with the addition of polymers. As these polymers have a stabilizing and thickening effect, they have the added advantage of increasing the mouth feel of the drink. The polymers were added at a concentration of 1 wt% to the solution so that the properties of the drinks were not altered.

The interaction of acids with the enamel results in the dissolution of the HA crystals to Ca2+, PO43‐, and OH‐ ions, causing surface softening of the enamel. Prolonged treatment of the enamel with acid leads to a continuous DE, resulting in an extensive loss of the enamel matrix [17]. The dissolution of the enamel on contact with an acidic solution depends on pH, acidic concentration and type, titrable acidity, and degree of saturation (DS) concerning HA [18,19]. Different preventive strategies were put forward to reduce the deleterious effects of soft drinks. Diet modification and altered composition of acidic drinks are emphasized as methods to reduce erosion. Various methods suggested to decrease the erosiveness of drinks are to increase their pH and add calcium- and/or phosphate-containing salts to increase DS. However, these may lead to lesser appealing drink taste and elevated microbiological spoilage affecting their market value [20]. Studies have reported a reduction in the erosiveness of beverages when modified with fluoride and citrate [19]. Agents that reduce the dissolution rate of HA fraction of enamel and dentin could be used in food products to prevent DE [14].

The food-approved polymers in acidic drinks are a favorable approach for reduction in erosion. Investigation into this aspect of tooth-friendly soft drinks is limited. In the present study, three different food-approved polymers were assessed for their anti-erosive properties. Pectin is a polysaccharide used in food products like jams, jellies, frozen foods, and more recently in low-calorie foods as a fat and sugar substitute. Apple pomace and orange peel are the two major sources of commercial pectin. The structure of pectin consists of homogalacturonan (HG) and type I rhamnogalacturonan (RG-I) areas. Pectin HG sections are composed of polygalacturonic acid residues that are partially methyl-esterified. Pectins having a percentage of methyl esterification (DM) greater than 50% are referred to as high methoxyl (HM) pectins, whereas those with less than 50% are referred to as low methoxyl (LM) pectins. LM pectins are beneficial for delivering medications, particularly in intranasal formulations, due to their ability to create gels when combined with Ca2+ ions. They undergo binding with Ca2+ ions or a solute under acidic conditions to create a gel-like substance. This characteristic can be useful in the decrease of tooth demineralization, as pectin establishes a link with Ca2+ ions in HA. Alginate is derived from aquatic organisms and is used in the food manufacturing and cosmeceutical sectors [21].

Polyguluronic acid is a compound formed by the esterification of certain carboxyl groups of alginic acid with propylene glycol. Additionally, certain carboxyl groups are neutralized using a suitable alkali, while others are left unreacted. The erosion-protective function of HA particles (Ca2+ ions) can be attributed to their chemical reaction with carboxyl groups. GA is a substance obtained from the twigs of *Acacia senegal* trees, and it serves as both an emulsifying agent and a stabilizer. It serves as a component in chocolate and confections and serves a crucial function in the carbonated beverage sector by facilitating the bonding of sugar to the beverage. The substance is composed of glycoproteins and polysaccharides and typically has little impact on the food or flavor of the beverage [22]. Glycyrrhetinic acid attaches to HA nanoparticles by electrostatic and hydrogen-bonding interactions with positively charged surface locations, primarily Ca2+ ions found on the enamel layer [23]. The creation of several layers due to hydrogen-bonding interactions between polymer molecules results in a decrease in enamel erosion [13].

This study is the first of its kind to employ an LIFS system to quantify tooth demineralization by acidic drinks. The main principle behind this technique is that when a photon from a laser falls on the tooth surface, it gets penetrated and absorbed by fluorophores present endogenously. These absorbed fluorophores re-emit lower energy photons in the form of characteristic LIF spectra. In the case of tooth demineralization, the biochemical structure and composition of samples will be altered, and this will be reflected in the fluorescence spectral shape and intensity. The variation in concentration of fluorophores occurs before structural changes in the tooth, enabling the detection of demineralization in an early stage [24].

Significant changes in fluorescence intensity could be appreciated even at 30 seconds of acidic exposure using the LIFS system. The polymer-modified carbonated drink solutions used in this study showed an increase in the intensity of fluorescence of enamel samples indicating a decreased demineralization than plain carbonated drink. The reason for this reduced erosion of food-approved polymer-added carbonated drinks could be the deposition of the polymer layer on the enamel surface [13]. A protective layer of polymer formation on the tooth surface was suggested as the reason for the reduced demineralization [25,26]. This phenomenon of protection is different from the usage of a remineralizing agent. In the shortest time of exposure of 30 seconds, all three polymers were suggested to be effective in decreasing the erosiveness of acidic drinks. GA-contained carbonated drink solution showed effectiveness against demineralization in a 30-second treatment. This result is contradictory to that of Barbour et al., who reported that polymer-modified citric acid solutions containing GA (pH 3.2; 0.02% polymer) did not affect the dissolution of artificial HA discs [14]. This might be because the present study used commercially available drinks and the chemical interaction of GA with its constituents could have increased its protectiveness. The pH of the GA-modified solution in the current study is 2.7, which is different from the polymer-modified citric acid solution.

An advantage of utilizing the LIFS technique for assessing erosive possibilities is its ability to accurately quantify enamel demineralization, even following only a short time of exposure. Prior research has demonstrated the feasibility of recognizing and tracking initial mineral depletion in primary teeth utilizing quantitative laser fluorescence (QLF) [27]. Borisova et al. [11] showed that laser-induced autofluorescence spectroscopy may effectively distinguish between nascent caries lesions and demineralized teeth. Ando et al. [28] observed a reduction in fluorescence signal intensity during demineralization, which was attributed to the degradation of the enamel layer's prism structure and alterations in its waveguide properties. HP was determined to exhibit greater efficacy with a 30-second exposure compared to prolonged durations of therapy. PGA demonstrated a substantial decrease in demineralization within 30 and 120 seconds. The study demonstrated that GA effectively reduced demineralization during periods of 30, 60, and 120 seconds, as seen by significant modifications in fluorescence intensity. The fluorescence intensity of the enamel samples exposed to plain carbonated drink exhibited a notable reduction as the duration extended. This suggests that the process of demineralization caused by a carbonated beverage becomes more pronounced as the duration of exposure advances.

The tooth specimens from the five groups were subjected to the test solutions for 120 seconds, and SEM micrographs were captured. When comparing the specimens subjected to carbonated drink with the distilled water sample, it was seen that the surface structure of the former exhibited a more uneven appearance. Additionally, the apertures of dentinal tubules were visible, indicating signs of erosion. The enamel specimens subjected to polymer-modified carbonated drink solutions had a notably smooth surface. The potential cause for the development of a smoother surface in enamel treated with polymer-infused solutions could be attributed to the relationship that exists between the HA particles and the polymer matrix. The presence of polymers and dissolved HA particles in the surface layer can serve as a layer of protection, preventing the erosive impact of carbonated beverages. The presence of polymers in HP, PGA, and GA solutions contributes to the development of a smooth texture on enamel through the process of adsorption. Li conducted a study where they used the tapping-mode atomic force microscopy (AFM) to observe the adsorption of xanthan gum on the enamel [29].

The occurrence of a prism structure in the SEM examination can be ascribed to enamel erosion, which is observed in the enamel samples exposed to a plain carbonated beverage. The decrease in the visibility of the prism pattern after treatment with polymer-modified solutions may be attributed to either the presence of a polymer layer that has been adsorbed or simply a result of the diminished erosion capability of polymers. The food polymers utilized in this investigation serve as agents that thicken and stabilize liquids, hence influencing their viscosity. Prior studies have documented the impact of HP, PGA, and GA on altering viscosity. The incorporation of polymers into an acidic solution can cause a rise in viscosity, which in turn can result in lowered ion movement and delayed dissolution kinetics. This can contribute to a reduction in demineralization [30]. The enamel, which underwent treatment with a solution containing additional pectin, exhibited a surface that was more seamless in comparison to the surfaces of the other two polymers.

The study limitations include the following: its important to acknowledge that this research was conducted in a laboratory setting using premolar teeth, rendering it an in vitro study. Such controlled conditions may not fully replicate the dynamic and multifaceted environment of the oral cavity, which includes factors like salivary flow and oral hygiene practices. While the study suggests the potential of polymer additives to mitigate enamel demineralization, its clinical applicability remains uncertain. Further research, particularly clinical trials involving human subjects, is necessary to evaluate the safety, feasibility, and effectiveness of incorporating these polymers into commercially available beverages as a preventive measure against DE.

## Conclusions

The incidence of tooth erosion is on the rise, particularly among young adults, as a result of the heightened consumption of acidic meals and soft drinks. This study demonstrates that incorporating food-grade polymers, specifically HP, PGA, and GA, into a carbonated beverage that is already on the market has the potential to decrease its erosive properties. The food polymers' characteristics mentioned can be observed through the quantitative evaluation of demineralization caused by an acidic challenge, utilizing laser fluorescence spectroscopy. Spectroscopic techniques are a rapid and accurate approach for quantifying enamel demineralization, facilitating early identification. Although all the polymers exhibited equivalent efficacy, HP demonstrated superior resistance to acidic challenge. The SEM pictures further demonstrated the protective impact of polymers by exhibiting a sleek surface morphology in the enamel that was exposed to solutions treated with polymers.
